# Perioperative Serum Calcium and Phosphorus Levels are Associated with Hospital Costs and Length of Stay after Major Abdominal Surgery

**DOI:** 10.3390/jcm7100299

**Published:** 2018-09-22

**Authors:** Tak Kyu Oh, Jihoon Jo, Ah-Young Oh

**Affiliations:** 1 Department of Anesthesiology and Pain Medicine, Seoul National University Bundang Hospital, Seongnam 13620, Korea; airohtak@hotmail.com (T.K.O.); 54272@snubh.org (J.J.); 2 Department of Anesthesiology and Pain Medicine, Seoul National University, Seoul 03080, Korea

**Keywords:** calcium, general surgery, malnutrition, phosphorus

## Abstract

This study aimed to identify an association between serum calcium (Ca) and phosphate (P) levels, tested during the pre- and postoperative period, with hospital costs and length of hospital stay (LOS) of patients who underwent major abdominal surgery. This retrospective cohort study analyzed the medical records of patients who underwent major abdominal surgery. A total of 3893 patients were included in the analysis, and multivariable linear regression analysis was performed. For a 1 mg/dL increase in preoperative Ca, total hospital costs decreased by 3997.9 dollars (coefficient: −3997.9, 95% confidence interval (CI): −4900.5, −30,953; *p*-value < 0.01), and for a 1 mg/dL increase in postoperative P, total hospital costs decreased by 702.5 dollars (coefficient: −702.5, 95% CI: −1274.5, −67.3; *p*-value = 0.03). Furthermore, for a 1 mg/dL increase in preoperative Ca, LOS decreased by 2.9 days (coefficient: −2.9, 95% CI: −3.7, −2.1; *p*-value < 0.01). For a 1 mg/dL increase in postoperative P, LOS decreased by 3.4 days (coefficient: −3.4, 95% CI: −4.2, −2.6; *p*-value < 0.01). This study suggested preoperative Ca and postoperative P could be useful indicators for the reduction of hospital costs and decrease in LOS from the perspective of enhanced recovery after surgery.

## 1. Introduction

Malnutrition is considered a significant health-related problem that leads to increased mortality, morbidity, and health costs [1]. In patients undergoing surgery, preoperative malnutrition can increase postoperative complications [2,3] and is known to increase postoperative mortality [4,5]. Therefore, evaluating the nutritional status of patients prior to surgery is important for predicting postoperative outcome, and various indices have been studied for effective evaluation [6,7].

Serum calcium (Ca) and phosphorus (P) play an important role in bone mineralization, membrane transport, energy production, and vascular function in the human body [8]. Considering that Ca and P levels are maintained by the gastrointestinal tract and the kidneys [8], deficiencies of Ca or P could be an indicator of underlying pathologic conditions, such as malnutrition, pancreatitis, sepsis or septic shock, acute kidney injury, or chronic kidney disease [9]. These pathologic conditions often develop in the perioperative period; thus they can be considered perioperative complications. In addition, bleeding from surgical procedures can lead to an electrolyte imbalance, and transfusion of stored blood using citrate is known to cause perioperative Ca deficiency [10]. Therefore, patient Ca or P deficiency could be closely related with increased costs or length of stay (LOS) after surgery. However, there has been no study analyzing potential associations between perioperative Ca and P levels and hospital costs or LOS after surgery.

Therefore, the purpose of this study was to identify an association between Ca and P levels, measured during the pre- and postoperative periods, with the overall hospital costs and LOS of patients who undergo major abdominal surgery.

## 2. Materials and Methods

Medical records of patients aged ≥19 years who underwent elective major abdominal surgery at a national university hospital from January 2010 to December 2017 were included. Major abdominal surgery was defined as all abdominal surgical procedures with surgery time >2 h and estimated blood loss >500 mL. Therefore, in addition to exploratory laparotomy, all general surgery for the gastrointestinal tract, hepatobiliary surgery, pancreatic surgery, surgery of the spleen and major vessels, obstetrics and gynecologic surgery including Cesarean section, and all urologic surgery for the genitourinary tract were included.

### 2.1. Total Medical Costs for Major Abdominal Surgery

In Korea, most patients are obligated to enroll in the National Health Insurance Service provided by the national government, and approximately two thirds of the necessary medical costs are covered by this health insurance system [11]. The central government subsidizes all medical costs for patients with very low income, and patients from abroad must pay all costs out-of-pocket. In this study, total costs were calculated as the sum of the payment made by the patients at discharge after recovering from surgery and the payment covered by the health insurance system. All data on costs were collected in Korean currency of Won (₩), which were converted according to a ratio of 1060 won to 1 U.S. dollar ($). Dollar was used as the ultimate unit of cost.

### 2.2. Measurements and Outcome

Patients’ demographic information (age, body mass index, sex), preoperative comorbidities, and surgery-related information were collected for analysis in this study. Preoperative comorbidities included preoperative hypertension, diabetes mellitus, history of ischemic heart disease and cerebrovascular disease, liver disease (hepatitis, liver cirrhosis, hepatocellular carcinoma), and cancer.

Surgery-related information included surgery time (h), estimated blood loss (mL), total relative value units (RVU), types of surgery (gastrointestinal tract surgery, hepato-biliary-pancreatic surgery, genitourinary surgery, obstetric and gynecologic surgery, major vascular surgery, and exploratory laparotomy or bleeding control), laparoscopy, and intraoperative remifentanil dosage (mg). Total RVUs reflect surgical complexity [12]. Total RVUs for each surgery represent the total of three component RVUs: one for physician work (36.1%), one for practice expenses (62.1%), and one for malpractice expenses (1.8%), and can be downloaded from the homepage of the Health Insurance Review and Assessment Service in South Korea (http://www.hira.or.kr/eng/main.do). We used the total RVUs, as updated in July 2018.

Preoperative laboratory testing was performed within at least 1 month prior to surgery and for all patients who were scheduled for elective surgery; Ca (mg/dL) and P (mg/dL) levels were included in this testing. In addition, postoperative routine laboratory testing was performed on postoperative day 0–1 for most patients who underwent major abdominal surgery; Ca and P levels were also included in this testing. If Ca and P levels were tested several times during the preoperative period, the most recent data prior to the surgery was defined as preoperative Ca or P. If Ca and P levels were tested several times during postoperative day 0–1, the first tested level was defined as postoperative Ca or P. LOS was measured as number of days from postoperative day 1.

The primary outcome of this study was to identify an association between pre- and postoperative Ca and P level, and total costs. Additionally, the association with LOS after surgery was identified.

### 2.3. Ethics

This retrospective cohort study was conducted in accordance with the ethical standards of the responsible institutional according to the guidelines of the Helsinki Declaration of 1975 as revised in 1983 (Approval number of institutional review board: B-1809/495-101). Considering the characteristics of a retrospective cohort design, which analyzes medical records of patients who completed treatment, the need for informed consent was waived.

### 2.4. Statistical Analysis

The baseline characteristics of patients are presented as mean with standard deviation and number with percentage. We analyzed the association between costs and LOS with each covariate using univariable linear regression analysis. In the univariable linear regression model, variables with *p*-value < 0.1 were included in the final multivariable linear regression analysis. The results of this linear regression analysis are presented as regression coefficients and 95% confidence intervals (CI). In addition, total costs in dollars and LOS after surgery according to pre- and postoperative Ca or P were presented through restricted cubic spline. All analyses were performed using IBM SPSS version 23.0 (IBM Corp., Armonk, NY, USA), and results with *p*-value < 0.05 were considered statistically significant.

## 3. Results

**Figure 1:**
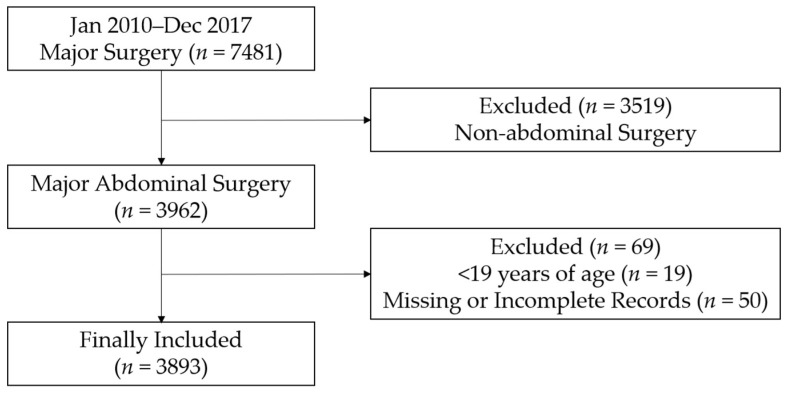
Flow chart for patient selection.

From January 2010 to December 2017, a total of 7481 patients underwent major surgery (surgery time > 2 h, estimated blood loss > 500 mL). Among these, 3519 patients who underwent non-abdominal surgery, 19 patients who were <19 years of age, and 50 patients with incomplete or missing medical records were excluded. As a result, a total of 3893 patients who underwent major abdominal surgery were included for the final analysis (Figure 1). The baseline characteristics of the patients are presented in Table 1. The LOS after surgery (day) of all patients was 14.6 ± 16.9, and the total hospital costs ($) were 16,807.3 ± 21,665.3.

### 3.1. Total Costs according to Pre- and Postoperative Calcium and Phosphorus Levels

**Figure 2:**
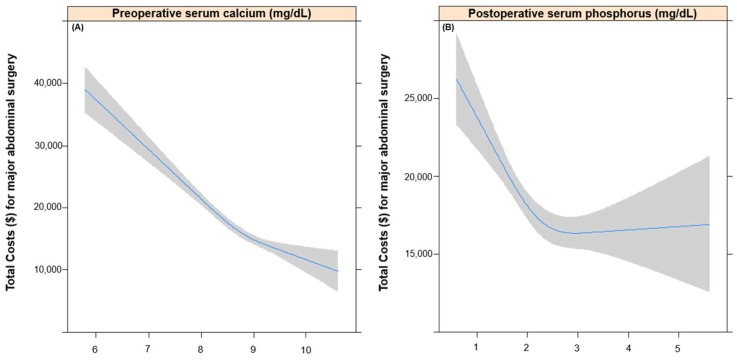
Relationship between total costs and preoperative calcium (**A**) and postoperative phosphorus (**B**).

Table 2 shows the results of univariable linear regression analysis for total costs. Variables selected in this univariable model were input into the multivariable linear regression analysis; these are shown in addition to Ca and P levels in Table 3. A 1 mg/dL increase in preoperative Ca was associated with a 3997.9 dollar decrease of total hospital costs (coefficient: −3997.9, 95% CI: −4900.5, −3095.3; *p*-value < 0.01), while preoperative P showed no association with total costs (*p*-value = 0.29). In addition, a 1 mg/dL increase in postoperative *p* was associated with a 1016.9 dollar decrease of total hospital costs (coefficient: −702.5, 95% CI: −1,274.5, −67.3; *p*-value = 0.03), while postoperative Ca showed no association with total costs (*p*-value = 0.98). Figure 2 shows the negative linear relationship between total costs and preoperative Ca (2A) and postoperative P (2B), which showed independent negative association with costs.

### 3.2. Length of Hospital Stay after Major Abdominal Surgery according to Pre- and Postoperative Calcium and Phosphorus Levels

**Figure 3:**
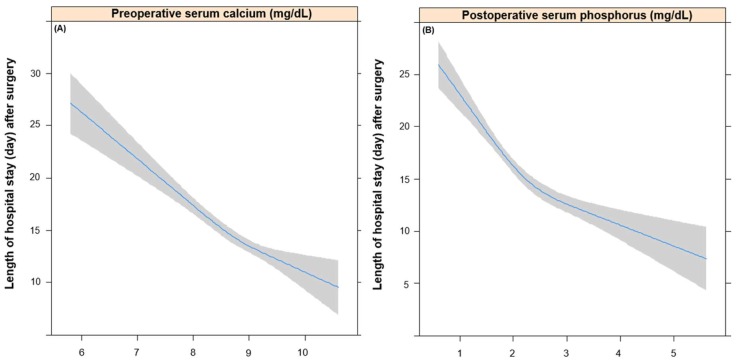
Relationship between length of hospital stay and preoperative calcium (**A**) and postoperative phosphorus (**B**).

Table 2 shows the results of univariable linear regression analysis for LOS (day) after surgery. Variables selected in this univariable model were input into the multivariable linear regression analysis; these are shown in addition to Ca and P levels in Table 3. A 1 mg/dL increase in preoperative Ca was associated with a 3.2 day decrease of LOS (coefficient: −3.2, 95% CI: −4.1, −2.4; *p*-value < 0.01), while preoperative P showed no association with LOS (*p*-value = 0.05). In addition, a 1 mg/dL increase in postoperative P was associated with a 3.3 day decrease of LOS (coefficient: −3.3, 95% CI: −4.0, −2.5; *p*-value < 0.01), while postoperative Ca showed no association with LOS (*p*-value = 0.34). Figure 3 shows the relationship between LOS and preoperative Ca (Figure 3A) and postoperative P (Figure 3B), which showed an independent negative association with LOS after surgery.

## 4. Discussion

This study included patients who underwent major abdominal surgery, and showed that preoperative Ca was negatively associated with hospital cost and LOS, while postoperative P was negatively associated with hospital cost and LOS. In other words, low preoperative Ca levels and low postoperative P levels are associated with increased total hospital costs and LOS. This study is meaningful as it is the first study to show the significance of Ca and P in perioperative nutritional management, particularly in patients undergoing major abdominal surgery.

Recently, the concept of enhance recovery after surgery (ERAS) has been introduced to reduce wasted medical costs and the LOS of patients receiving surgery [13]. From the perspective of ERAS, this study suggested that Ca or P is a useful indicator for clinicians in predicting fast recovery after major abdominal surgery. Ca is a useful indicator in the preoperative period, while P is useful in the postoperative period. A recent study, reported by the ERAS society [14], perioperative nutritional support in cancer patients is very important in cancer surgery, because perioperative malnutrition in surgical patients is closely associated with prolonged recovery [15]. Considering that there is no optimal assessment tool of nutritional status in surgical patients except albumin or body mass index [16], our study suggested that Ca or P might be indicators for assessing the nutritional status of surgical patients.

An important finding of this study is that Ca, not P, is associated with reduction of hospital costs and LOS after major abdominal surgery. This can be explained by two reasons. First, preoperative sarcopenia, as well as malnutrition, is known to be an important element for the ERAS program, predicting the increase in postoperative complications or prolonged LOS. Sarcopenia, a form of muscle loss, is known to have a strong inverse association with daily Ca intake [17], lower preoperative Ca might be an indicator of sarcopenia in patients who underwent major abdominal surgery in this study. Secondly, deficiency of micronutrients, such as Ca, is known to induce immune deficiency [18] and, the results of this study may indicate that Ca deficiency during the preoperative period could lead to reduced postoperative immunity, thereby increasing LOS or hospital costs.

The finding that P rather than Ca, influences the postoperative period by being negatively associated with increased hospital costs or LOS could be understood for other reasons. Postoperative hypophosphatemia is common, which is known to be a result of hemodilution caused by bleeding or fluid administration during surgery [19]. Therefore, the degree of decrease in postoperative P may reflect bleeding or positive fluid balance during surgery. Considering that positive fluid balance increases LOS and postoperative complications, postoperative hypophosphatemia may indirectly reflect perioperative positive fluid hypophosphatemia [20]. Postoperative severe hypophosphatemia may develop in postoperative patients due to alcohol withdrawal, diabetic ketoacidosis, refeeding syndrome, and severe respiratory alkalosis [21], which are factors that can increase hospital costs and LOS. Moreover, postoperative hypophosphatemia can itself cause muscle weakness [22], which would have a negative impact on early rehabilitation or recovery. Therefore, postoperative hypophosphatemia is an index that can directly reflect positive fluid balance during surgery, postoperative complications, and delay in recovery. However, as the present study used only the first P level in analysis, a longitudinal analysis using P should be performed in the future.

This study has some limitations. First, as a limitation of a retrospective cohort design, selection bias may be present, and the quality of data may be lower than data of a prospective study. Second, the findings may not be generalizable to other populations, as this study was conducted in a single hospital. Lastly, Ca and P tests were not conducted at the same time before and after surgery in all patients. However, despite these limitations, the findings of this study are useful, as this is the first study to show an association between perioperative Ca and P levels with total hospital costs and LOS after major abdominal surgery.

## 5. Conclusions

This study showed a negative association between preoperative Ca and postoperative P levels with total hospital costs and LOS, respectively, in patients who underwent major abdominal surgery. This finding suggested preoperative Ca and postoperative P could be useful indicators for the reduction of hospital costs and decrease in LOS from the perspective of ERAS.

## Figures and Tables

**Table 1:** Characteristics of patients who received major abdominal surgery in 2010–2017.

Variables	Total (3893)	Mean (SD)
Age, year		58.5 (14.6)
Body mass index, kg m^-2^		23.9 (3.6)
Sex: male	2079 (53.4%)	
Preoperative comorbidities		
Hypertension	1096 (28.2%)	
Diabetes mellitus	211 (5.4%)	
Ischemic heart disease	75 (1.9%)	
Cerebrovascular disease	84 (2.2%)	
Chronic kidney disease	208 (5.3%)	
Liver disease (Hepatitis, LC, HCC)	362 (9.3%)	
Cancer	300 (7.7%)	
Information regarding surgical procedures		
Surgery time, hour		5.3 (2.4)
Estimated blood loss, mL		1310.5 (2154.2)
Total RVUs ^a^		29,485.9 (33,927.1)
Intraoperative remifentanil dosage, mg		1.0 (1.8)
Length of hospital stay, day		14.6 (16.9)
Type of surgery		
Gastrointestinal tract surgery	895 (23.0%)	
Hepato-biliary-pancreatic surgery	971 (24.9%)	
Genitourinary surgery	689 (17.7%)	
Obstetric and gynecologic surgery	935 (24.0%)	
Major vascular surgery	87 (2.2%)	
Exploratory laparotomy or bleeding control	316 (8.1%)	
Laparoscopic surgery	602 (15.5%)	
Total hospital costs, dollar		16,807.3 (21,665.3)
Preoperative serum Ca, mg/dL		8.7 (0.7)
Preoperative serum P, mg/dL		3.5 (0.8)
Postoperative serum Ca, mg/dL		6.2 (2.6)
Postoperative serum P, mg/dL		2.3 (0.8)

SD: standard deviation; LC: liver cirrhosis; HCC: hepatocellular carcinoma; RVU: relative value unit; Ca: calcium; P: phosphorus; ^a^: Total RVUs for each surgery represent the total of three component RVUs: one for physician work (36.1%%), one for practice expenses (62.1%), and one for malpractice expenses (1.8%), and can be downloaded from the homepage of the Health Insurance Review and Assessment Service in South Korea: http://www.hira.or.kr/eng/main.do. We used the total RVUs, updated in July 2018. Total RVUs of surgery are often used for adjustment of surgical complexity.

**Table 2:** Univariable linear regression analysis for total costs ($) and length of hospital stay (day) after major abdominal surgery.

Characteristics	Total Costs ($)		Length of Hospital Stay (day)	
	Coefficient (95% CI)	*p*-Value	Coefficient (95% CI)	*p*-Value
Sex: male (vs. female)	5393.8 (4035.3, 6752.3)	<0.01	3.2 (2.2, 4.3)	<0.01
Age, year	125.7 (78.9, 172.5)	<0.01	0.2 (0.1, 0.2)	<0.01
Body mass index, kg/m^2^	−248.7 (−442.2, −55.2)	0.01	−0.4 (−0.5, −0.2)	<0.01
Hypertension	1496.8 (−19.2, 3012.7)	0.05	3.4 (0.2, 4.6)	<0.01
Diabetes mellitus	4440.6 (1429.5, 7451.8)	<0.01	2.4 (0.2, 4.8)	0.05
Cerebrovascular disease	6245.1 (1295.4, 11,194.8)	0.01	10.5 (6.6, 14.3)	<0.01
Ischemic heart disease	8967.5 (4289.8, 13,645.3)	<0.01	4.3 (0.6, 8.0)	0.02
Chronic kidney disease	21,735.9 (18,765.0, 24,706.8)	<0.01	6.8 (4.4, 9.2)	<0.01
Liver disease	19,687.8 (17,423.3, 21,952.4)	<0.01	2.0 (0.1, 3.8)	0.04
Cancer	1936.2 (−620.5, 4493.0)	0.14	2.4 (0.4, 4.5)	0.02
Surgery time, hour	2295.7 (2026.6, 2564.7)	<0.01	1.9 (1.6, 2.1)	<0.01
Estimated blood loss, 100 mL	330 (304, 362)	<0.01	1.0 (1.0, 1.1)	<0.01
General surgery (vs. non-GS)	13,683.2 (12,368.0, 14,998.4)	<0.01	7.6 (6.5, 8.6)	<0.01
Laparoscopy	−5005.3 (−6884.0, −3126.6)	<0.01	−4.1 (−5.5, −2.6)	<0.01
Total RVUs ^a^, 10000 points	2544.5 (2380.3, 2708.7)	<0.01	0.53 (0.38, 0.68)	<0.01
Intraop rmFTN dosage, 1 mg	−139.3 (−479.0, 200.4)	0.42	0.96 (0.68, 1.23)	<0.01

Variables with *p* < 0.1 were included multivariable linear regression analysis in Table 3. ^a^: Total RVUs for each surgery represent the total of three component RVUs: one for physician work (36.1%), one for practice expenses (62.1%), and one for malpractice expenses (1.8%), and can be downloaded from the homepage of Health Insurance Review and Assessment Service in South Korea: http://www.hira.or.kr/eng/main.do. We used the total RVUs, updated in July 2018. Total RVUs of surgery are often used for adjustment of surgical complexity. Ca: Calcium; P: Phosphorus; GS: General surgery. Intraop rmFTN: Intraoperative remifentanil.

**Table 3:** Linear regression analysis for total costs ($) and length of hospital stay (day) after major abdominal surgery according to perioperative serum Ca and P.

	Total Costs ($)		Length of Hospital Stay (day)	
Variables	Coefficient (95% CI)	*p*-Value	Coefficient (95% CI)	*p*-Value
Unadjusted				
Preoperative Ca	−6190.0 (−7132.9, −5247.1)	<0.01	−3.7 (−4.5, −3.0)	<0.01
Preoperative P	−672.9 (−1594.6, 248.8)	0.15	−0.6 (−1.2, 0.1)	0.11
Postoperative Ca	658.6 (385.2. 932.0)	<0.01	0.2 (0.0, 0.4)	0.07
Postoperative P	−2076.3 (−3008.1, −1144.5)	<0.01	−3.7 (−4.4, −3.0)	<0.01
Adjusted				
Preoperative Ca	−3997.9 (−4900.5, −3095.3)	<0.01	−2.9 (−3.7, −2.1)	<0.01
Preoperative P	−512.4 (−1451.6, 426.7)	0.29	0.6 (−0.3, 1.4)	0.18
Postoperative Ca	3.8 (−247.9, 255.4)	0.98	−0.2 (−0.4, 0.0)	0.098
Postoperative P	−702.5 (−1274.5, −67.3)	0.03	−3.4 (−4.2, −2.6)	<0.01

All covariates of *p* < 0.1 in each univariable linear regression models (Table 2) were included in multivariable linear regression analysis.
